# Energy-saving pretreatments affect pelagic *Sargassum* composition and DNA metabarcoding reveals the microbial community involved in methane yield

**DOI:** 10.1371/journal.pone.0289972

**Published:** 2023-08-17

**Authors:** Enrique Salgado-Hernández, Ángel Isauro Ortiz-Ceballos, Alejandro Alvarado-Lassman, Sergio Martínez-Hernández, Erik Samuel Rosas-Mendoza, Jesús Bernardino Velázquez-Fernández, Ana Elena Dorantes-Acosta

**Affiliations:** 1 Instituto de Biotecnología y Ecología Aplicada (INBIOTECA), Universidad Veracruzana, Xalapa, Veracruz, Mexico; 2 División de Estudios de Posgrado e Investigación, Tecnológico Nacional de México/Instituto Tecnológico de Orizaba, Orizaba, Veracruz, Mexico; 3 CONACYT-Tecnológico Nacional de México/Instituto Tecnológico de Orizaba, Orizaba, Veracruz, Mexico; 4 CONACYT-Centro de Investigación y Asistencia en Tecnología y Diseño del Estado de Jalisco A.C., Guadalajara, Jalisco, Mexico; Tsinghua University, CHINA

## Abstract

*Sargassum* spp. flood the Caribbean coastline, causing damage to the local economy and environment. Anaerobic digestion (AD) has been proposed as an attractive option for turning macroalgae into valuable resources. *Sargassum* spp. has a complex composition that affects the microbial composition involved in AD which generates a low methane yield. This study aimed to improve the methane yield of pelagic *Sargassum*, using different energy-saving pretreatments and identifying the microbial community associated with methane production. We applied different energy-saving pretreatments to algal biomass and assessed the methane yield using a biomethane potential (BMP) test. The microbial communities involved in the AD of the best- and worst-performing methanogenic systems were analyzed by high-throughput sequencing. The results showed that pretreatment modified the content of inorganic compounds, fibers, and the C:N ratio, which had a strong positive correlation with BMP. The water washing pretreatment resulted in the best methane yield, with an increase of 38%. DNA metabarcoding analysis revealed that the bacterial genera *Marinilabiliaceae_uncultured*, *DMER64*, *Treponema*, and *Hydrogenispora*, as well as the archaea genera *Methanosarcina*, *RumEn_M2*, *Bathyarchaeia*, and *Methanomassiliicocus*, dominated the microbial community with a high methane yield. This study is the first to demonstrate the microbial community structure involved in the AD of *Sargassum* spp. The pretreatments presented in this study can help overcome the limitations associated with methane yield.

## 1. Introduction

Since 2011, unusually large quantities of pelagic *Sargassum* have reached the Mexican Caribbean coast [1]. Two species (one with two morphological forms) of holopelagic *Sargassum* have been identified: *S*. *fluitans III*, *S*. *natans I*, and *S*. *natans VIII* [2]. The beaching of massive quantities of *Sargassum* spp. results in the accumulation of decomposing material that stains the coastal waters a murky brown. This is damaging not only to the tourism industry but also to the long-term health of coastal ecosystems [3]. On tourist beaches, macroalgae have been removed from the beaches and sea. However, these have been disposed of in areas that are not adequately prepared to prevent leachate from leaching into the aquifer [1]. Therefore, it is necessary to find a suitable use for the large volumes of *Sargassum* biomass. Researchers have explored the valorization of these macroalgae for biofuel generation and have primarily focused on the AD to produce biogas [4, 5]. Biogas production from brown algae may have a better potential energy output than biohydrogen, bioethanol, and biodiesel production. Also, the methane yields data of brown algae are higher than the terrestrial biomasses from sugar crops and lignocellulosic biomasses except for organic wastes; besides, the fact that it does not need to be cultivated makes biofuel production viable [6]. However, there are currently few studies on AD of these invasive species and published results report different biomethane potentials (BMP) [7–10]. In all cases, these values represent less than 40% of the theoretical biomethane potential (TBMP).

Low methane yields have been attributed to the compositional characteristics of *Sargassum* spp. that include a high content of minerals, ash, polyphenols, and high levels of insoluble fibers [7, 9]. These factors, together with the low carbohydrate content, which generates low C: N ratios, have been cited as the main reasons for the low methane yield of *Sargassum* spp. [11]. Brown macroalgae by themselves present high content of mineral salts [12], including mainly light metal ions, such as sodium, potassium, calcium, and magnesium [13]. It has been reported that at high concentrations of sodium, methane yield begins decreasing [14]. Because of this, different pretreatments to improve the solubilization of biomass and reduce inhibitory compounds, such as mineral salts, have been assessed on several species of macroalgae [15]. For pelagic *Sargassum* spp., Tapia-Tussell et al., used a fungal pretreatment and achieved a BMP of 104 mL CH_4_ g^-1^ VS [9]. While Thompson et al., used hydrothermal pretreatments and achieved a BMP of 116 mL CH_4_ g^-1^ VS [10]. However, the transfer of these technologies from laboratory to industrial scale is hampered by high capital investment and energy inputs that could not be offset by methane yields. Because of this, continuous efforts are currently being made to optimize pretreatments and achieve better yields at lower cost. Freshwater washing is a low-energy pretreatment method that is commonly employed in macroalgae biofuel development. Washing has been used to remove inert contaminants and salts that can hinder methane generation in high quantities [15]. Bruhn et al. [16] suspended the algae in water for 24 h and succeeded in diluting the concentration of salts and remove sand and gravel, which interfere with the measurement of biomass and methane yield. Tabassum et al. [17] showed that washing with hot water at 40°C has an advantage over washing with cold water, removing a higher percentage of ash and improving the volatile solids (VS) content, which improves the biomethane yield. In addition, the use of chemical solutions such as heating with water and dilute hydrochloric acid [18] and washing with Milli-Q nanopure water and different acid solutions [19] have been successfully tested for the removal of minerals and some inhibitory compounds in algae. In the case of these two *Sargassum* species, washing is a common method before AD. However, its effect on methane yield and chemical composition has not been evaluated.

In addition, there is little information on the influence of macroalgal composition on the microbial community in the AD process. It is known that different bacterial and archaeal consortia shape the biomass AD, which generates complex microbial communities. These microbial communities change based on the type of substrate [20] and their diversity increases with the chemical complexity of the substrate [21]. Therefore, it is necessary to understand the diversity of the microbial community that drives AD of *Sargassum* spp.

This study investigated the use of various low-energy pretreatments based on water and chemical solutions, as well as the microbial community involved in high and low methane yields. The results showed an effect on methane production, substrate biodegradability, and chemical composition, which have not been previously reported. The effects of *Sargassum* on the diversity of bacteria and archaea have also been reported. To the best of our knowledge, this is the first study to describe the microbial composition that performs pelagic *Sargassum* degradation in the AD process. The objectives of this study were: (i) to improve the biomethane potential of *Sargassum* spp. by improving its compositional characteristics using different energy-saving pretreatments and (ii) to identify the microbial community structure associated with biomethane yield.

## 2. Materials and methods

### 2.1 Biomass and inoculum collection

*Sargassum* spp. biomass was collected in Playa del Carmen (20°37’19.99’’N, 87°4’9.98’’W), Quintana Roo, Mexico. Sand and other natural contaminants attached (plastics, shells, feathers, and other algae) to the *Sargassum*, were removed manually. Samples were transported to the laboratory in a cooler at room temperature for 4 hours and stored in resealable plastic bags at -4°C for later use.

The inoculum was obtained from a pilot-scale reactor operating at ambient temperature and fed with the liquid fraction of municipal organic solid waste (LFMOSW), located at the Instituto Tecnológico de Orizaba (Veracruz, Mexico). The inoculum was incubated at 35°C under anaerobic conditions for 30 days and LFMORS was fed as the substrate to increase the microbial population and ensure methane production. The inoculum presented pH, total solids (TS), volatile solids (VS), and ash contents of 7.9 ±0.8, 4.15 ±0.08%, 60.62 ±9.9% TS, and 39.4 ±9.9% TS, respectively. Before biochemical methane potential testing, a sludge sample was taken for microbial analysis.

### 2.2 Pretreatments of *Sargassum* biomass

*Sargassum* samples were subjected to three different pretreatments to improve the composition of *Sargassum* biomass. The three different pretreatments are described below:

#### 2.2.1 Water washing (WW)

The wet biomass was manually washed with fresh water at a constant flow (0.920 L min^-1^) for 2 min at room temperature (25°C) to remove sand and other contaminants and allowed to drain for 5 min.

#### 2.2.2 Soaking + Warm water washing (S+W)

The wet biomass was immersed in fresh water at a ratio of 1: 4 (w: v) at room temperature for 24 h. Followed by a second wash with warm water at 40°C for 3 min and allowed to drain for 5 min. This method was modified based on Bruhn et al. [16] and Tabassum et al. [17].

#### 2.2.3 Chemical soaking (CS)

Two hundred grams of wet biomass were weighed and immersed in 400 mL of 2% formaldehyde (FHD) for a period of 16 h. The solution was drained, filtered, and rinsed with distilled water. Next, 500 mL of 0.2 M HCl was added for 24 h and again the samples were rinsed with distilled water. Finally, they were allowed to drain for 3 min. This method was modified from Fertah et al. [22]. The treatment was conducted at room temperature.

Samples of *Sargassum* biomass without pretreatment (as received), referred to as untreated (UT), were used. Finally, after pretreatments, samples were dried at 60°C to ~10% moisture and manually ground using ceramic mortar with a pestle into powder with a particle size ≤1 mm. The powdered *Sargassum* biomass was stored in resealable plastic bags at room temperature for further analysis and evaluation of BMP.

### 2.3 Biochemical methane potential (BMP) testing

The test to evaluate the BMP was established according to Holliger et al., [23]. The bioreactors consisted of 120 mL serum bottles with a working volume of 75 mL. The untreated (UT) and pretreated (WW, S+W, CS) samples were added to each bioreactor as substrate. Powdered cellulose (Sigma-Aldrich) was used as a positive control to ensure the accuracy of the tests. Inoculum-only blanks (negative control) were also prepared to measure the contribution of biogas from the inoculum. The inoculum to substrate ratio was 2: 1 according to VS. The pH values of the cultures were adjusted to 7.2 ±0.1, at the beginning of the experiment. All bottles were hermetically sealed with butyl rubber stoppers and aluminum caps. The headspace was purged with nitrogen gas, purity 99.5–100% (Praxair México S. de R.L. de C.V) for 3 min to reach anaerobic conditions. The bottles were incubated at 32°C until the daily methane production for three consecutive days was <1% of the accumulated volume. During this period, the bottles were shaken daily for 60 s. The volume of biogas was measured at regular intervals using a 5–60 mL glass syringe with a valve and luer-lock system. The biogas produced by the bioreactors was corrected with the biogas produced by the blank. The results were presented as the volume of gas (mL) at standard conditions (273 K and 1 atm) times the mass (g) of aggregated VS. The experiment was performed in triplicate (n = 3).

### 2.4 Analytical methods

Biogas composition was analyzed on a gas chromatograph (Buck Scientific 310, Norwalk, USA) with a thermal conductivity detector (TCD) equipped with a 6-inch long, 0.25-inch diameter CTR-I column. Helium was used as the carrier gas at 70 psi. The column temperature was 36°C, and the detector temperature was 121°C. TS, VS, and ash of the *Sargassum* and inoculum samples were measured according to Standard Methods [24]. The pH was measured using a Thermo Scientific™ Orion™ Versa star meter (Waltham, USA). The total phenol content (CFT) was analyzed by the Folin-Ciocalteu method using a UV-Vis spectrophotometer (Shimadzu, UV 1280, Kyoto, Japan). The analysis of carbon, hydrogen, and nitrogen content of untreated and pretreated biomass samples was analyzed using the elemental analyzer (PerkinElmer Series II CHNS/O Analyzer 2400, Waltham, USA). The total sulfur (S) was quantified by turbidimetry with gum arabic as a stabilizer, using a UV/Vis spectrophotometer (Thermo Scientific™ Spectronic 200, Waltham, USA). The oxygen content was estimated by difference. All the above analyses were performed in triplicate.

Cellulose, hemicellulose, and lignin content were determined by detergent fiber analysis [neutral detergent fiber (NDF), acid detergent fiber (ADF), acid detergent lignin (ADL)] on an ANKOM 200 fiber analyzer (ANKOM Technology, Fairport, NY, USA). The content of the major mineral salts was determined using a flame photometer, Corning 410 (Sherwood Scientific Ltd., Cambridge, UK) for Na and K, and an atomic absorption spectrometer, Varian 240FS (Agilent Technologies, Inc., Santa Clara, USA) for Ca and Mg. The above analyses were performed in duplicate.

### 2.5 Theoretical energy value and anaerobic biodegradability index

To calculate the theoretical biomethane potential (TBMP) and the empirical formula (C_n_H_a_O_b_N_c_) of pretreated and untreated *Sargassum* spp. biomass, the Buswell equation, and the Boyle equation were used [25, 26]. The Biodegradability Index (BI) was calculated as the ratio of BMP and TBMP as the percentage of TBMP achieved by the feedstock at the end of the digestion period.

The higher heating value (HHV) was calculated according to the modified Dulong formula described by Nizami et al. [27], and the lower heating value (LHV) was converted from the HHV as described by Deng et al. [28].

### 2.6 Microbial community analysis

#### 2.6.1 DNA extraction

Samples (1 mL) were taken from the inoculum and the WW and CS systems at the end of BMP tests, which were selected as exemplars of high and low methanogenic performance. Samples were centrifuged at 12 000 x g for 3 min to remove the liquid. DNA from the sludge was extracted using the DNeasy PowerSoil kit from QIAGEN (Hilden, Germany) according to the manufacturer’s instructions. DNA concentration, quality, and purity were estimated using agarose gel electrophoresis (1%) and with a Nanodrop^TM^ 2000 UV-Vis spectrophotometer (Thermo Scientific, Waltham, USA).

#### 2.6.2 16S rRNA gene metabarcoding and bioinformatics analysis

Once the quality of the DNA samples was assured, PCR amplification of selected regions was conducted using specific primers connected with barcodes. Hypervariable regions V4-V5 of the 16s rRNA gene for Bacteria were amplified using primer set 515F (5 ′-GTGCCAGCMGCCGCGGTAA-3 ′) and 907R (5′-CCGTCAATTCCTTTCTTTGAGTTT-3 ′). Similarly, regions V4-V5 of the 16s rRNA gene for Archaea were amplified using the primer set Arch519F (5’-CAGCCGCCGCGGGGTAA-3’) and Arch915R (5’-GTGCTCCCCCCCGCCAATTCCT-3’). PCR products with appropriate sizes were selected by 2% agarose gel electrophoresis. The same amount of PCR products from each sample was pooled, end-repaired, A-tailed, and ligated with Illumina adapters. Libraries were sequenced on the Illumina NovaSeq 6000 platform by Novogene Co (Beijing, China). After sequencing, forward and reverse reads with barcoded and primers removed were analyzed with QIIME2 software. Demultiplexed reads were denoised and assigned to amplicon sequence variants (ASVs) using the DADA2 algorithm. Taxonomic assignment was based on classifiers trained on the V4-V5 hypervariable region extracted from the SILVA 138 99% 16S sequence database. The confidence threshold for limiting taxonomic depth was set to 0.97.

### 2.7 Statistical analysis

A one-way analysis of variance (ANOVA) was performed to investigate the effect of pretreatments on biomass composition (ash content, total phenols, and minerals) and PBM at a 95% confidence interval limit. Statistical analyses were performed with the R programming language using RStudio version 1.3.1093 [29]. Spearman rank correlation was conducted to correlate the compositional characteristics of pretreated biomass with BMP and BI using IBM SPSS statistics 27.

## 3. Results and discussion

### 3.1 Effect of pretreatments on the composition of *Sargassum* spp.

The compositional analysis, along with the energy content of untreated and pretreated *Sargassum* spp. biomass are presented in Table 1.

**Table 1:** Effect of pretreatments on compositional characteristics of *Sargassum* spp. UT = Untreated; WW = Water washing; S+W = Soaking + Warm water washing; CS = Chemical soaking.

	UT	WW	S+W	CS
*Proximal analysis (n = 3)*				
TS (%)	89.1 ±0.8	89.9 ±0.06	86.3 ±0.05	91.8 ±3.4
VS (%)	44.7 ±0.7	67.3 ±1.4	64.0 ±0.5	70.2 ±3.6
Ash (%)	44.2 ±0.9	22.5 ±1.4	22.2 ±0.5	21.6 ±1.8
VS: ST ratio	0.50	0.75	0.74	0.76
A: V ratio*	0.98	0.33	0.35	0.31
*Ultimate analysis (% TS) (n = 3)*				
C	21.9 ±2.4	33.4 ±0.4	34.6 ±1.7	37.6 ±1.3
H	2.2 ±0.5	4.9 ±0.06	4.9 ±0.3	5.6 ±0.2
O	24.5 ±3.3	35.0 ±0.6	32.3 ±2.0	30.3 ±1.6
N	1.7 ±0.4	1.5 ±0.2	2.0 ±0.13	2.8 ±0.3
S	0.33 ±0.03	0.29 ±0.01	0.31 ±0.03	0.32 ±0.03
C: N ratio	13.2 ±1.6	22.2 ±2.5	17.0 ±1.6	13.6 ±1.2
*Structural composition (% TS) (n = 2)*				
Cellulose	3.7 ±0.60	13.2 ±0.20	12.0 ±0.89	15.2 ±1.5
Hemicellulose	8.2 ±0.59	10.1 ±1.20	11.9 ±0.56	8.0 ±0.90
Lignin	7.5 ±0.85	10.7 ±0.60	11.9 ±0.90	28.6 ±3.2
TPC**	4.3 ±0.2	4.7 ±0.07	3.5 ±0.16	1.6 ±0.18
*Energy content*				
HHV (kJ·g^-1^ VS)*	6.2	12.1	13.0	15.3
LHV (kJ·g^-1^ VS)*	5.5	10.8	11.7	13.9
TBMP (mL CH_4_·g^-1^ VS) *	335.8	424.8	452.7	503.8
Chemical formula	C_1.8_H_2.15_O_1.53_N_0.12_	C_2.8_H_4.9_O_2.18_N_0.10_	C_2.9_H_4.9_O_2.02_N_0.15_	C_3.13_H_5.6_O_1.9_N_0.19_

*A: V = Ratio of ash to volatile solids; HHV = Higher heating value; LHV = Lower heating value; TBMP = Theoretical biomethane potential.

**TPC = Total phenol content expressed as gallic acid equivalents (GAE) per gram of dry weight. Data are mean ± SD.

Proximate analysis of the untreated samples (as received) showed a high ash content of 43.9 ±1.3% and VS content of 44.7 ±0.9%. These results generated a high ash: volatile solid ratio (A: V) of 0.98, indicating a high inorganic material content. According to Tabassum et al. [17], high A: V ratios can inhibit AD. Similarly, the ratio of volatile solids to total solids (VS: TS) was 0.50, which represents the organic matter content in the sample. However, after pretreatment, the ash content was significantly affected (p <0.001) and representing a decrease of approximately 50%. The VS: TS ratio increased to 0.75, 0.74, and 0.76 for WW, S+W, and CS, respectively. No significant differences were found between the treatments (p >0.05). Positive effects of different pretreatments on the reduction in ash content have been reported. Diaz et al. [19] tested different demineralization pretreatments of *Sargassum* spp. using different chemical solutions and all successfully decreased the amount of ash. Moreover, washing in freshwater has been successfully reported to reduce ash content [30].

The ultimate analysis provided information on the relative percentages of the major elements present in the samples, typically carbon (C), hydrogen (H), nitrogen (N), sulfur (S), and oxygen (O). As shown in Table 1, the elemental composition changed after the pretreatments, resulting in a change in the C:N ratio. The C: N ratio of the untreated samples (13:1) was lower than those reported by Milledge et al. [7] and Thompson et al. [10] for *Sargassum* spp. A high C: N ratio indicates the accumulation of carbohydrates, which can be easily degraded during AD, whereas a low C: N ratio indicates a high protein content, resulting in excessive ammonia production, which causes toxicity to the methanogenic community [12]. After pretreatment, WW managed to improve the C: N ratio, reaching a value of 22:1, followed by S+W with a value of 17:1. While chemical soaking (CS) generated a low C: N ratio of 13:1, similar to that of the UT samples. The increase in the C: N ratio in the WW and S+W samples was attributed to the increase in the percentage of organic elements, mainly carbon, whereas nitrogen did not show a change after these pretreatments. In the CS samples, in addition to carbon, an increase in the nitrogen content was observed, which could lead to a low C: N ratio. According to these results, only WW pretreatment would be suitable for AD since the optimum C: N ratio for microbial growth is known to be 20–30:1. However, it is reported that the optimum C: N ratio can vary with algal species, from 14:1 to 30:1 [7].

**Fig 1:**
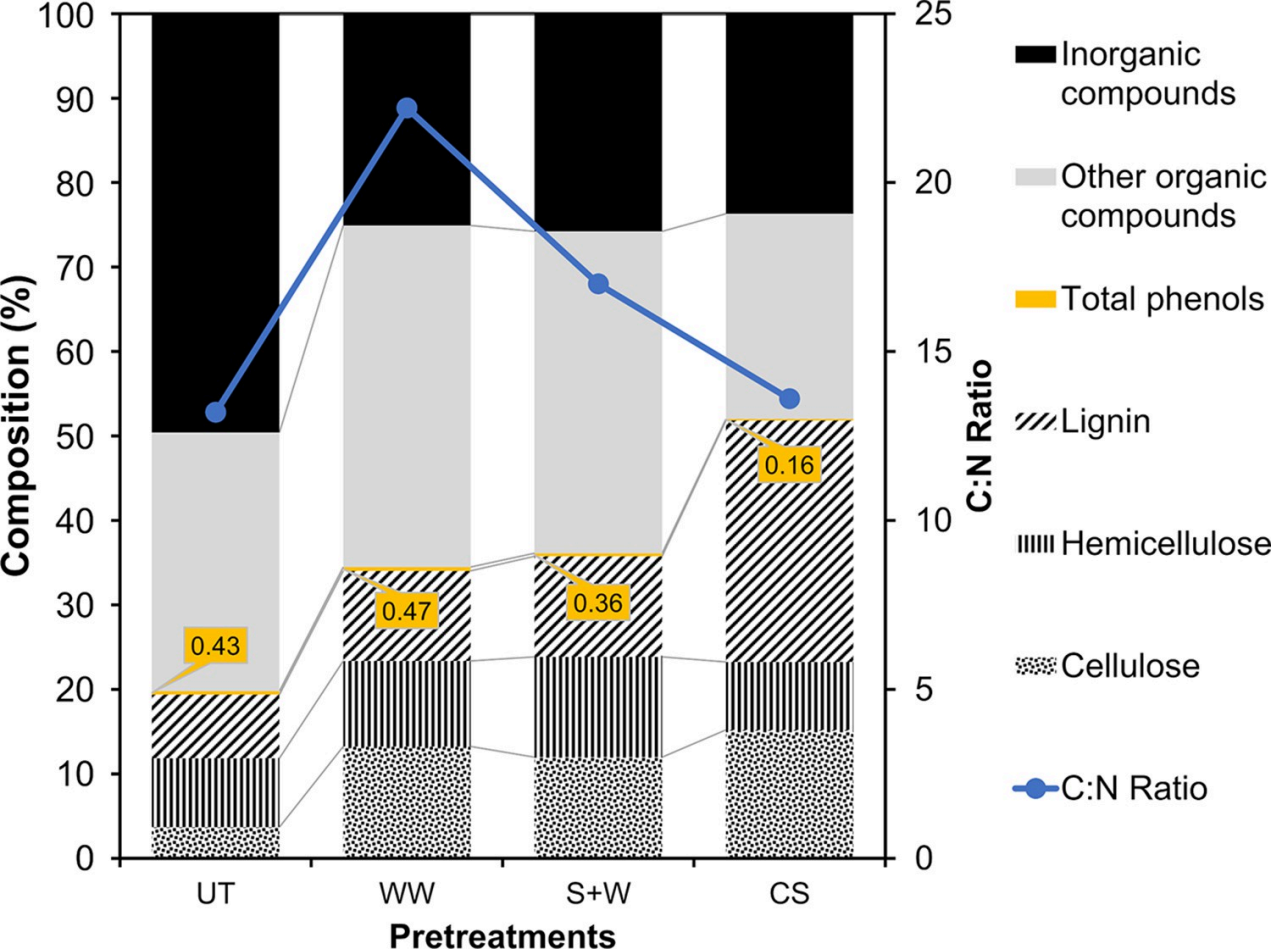
Changes in the compositional characteristics (% dry weight) of *Sargassum* spp. by the effect of pretreatments. UT = Untreated, WW = Water washing, S+W = Soaking + Warm water washing, CS = Chemical soaking.

The amounts of cellulose, hemicellulose, and lignin in UT biomass were 3.7 ±0.6, 8.2 ±0.6, and 7.5 ±0.8%, respectively. These results show that approximately 40% of the organic compounds in the algal biomass were composed of fibers. Brown macroalgae are characterized by a low lignin content and a high carbohydrate content, which makes them an attractive feedstock for AD [31]. However, the presence of lignin-like materials and lignified tissues in *Sargassum* spp. has been reported [32]. In previous studies, Salgado-Hernández et al. [33] reported a lignin content of 16.8%, while Tapia-Tussell et al. [9] reported a lignin content of 15.6%. Fig 1 shows the fiber content as a part of the organic matter compared to the inorganic components after pretreatments. It is possible to observe how the cellulose content increased threefold with WW and S+W due to ash removal. Hemicellulose and lignin contents did not change after pretreatment. However, CS pretreatment increased the fiber content by up to four times compared with UT. Mainly, the lignin content more than doubled compared to the other pretreatments. This result can be attributed not only to the removal of inorganic compounds, but also to the reduction of soluble carbohydrates.

The total phenol content (TPC) was 4.3 ±0.27 mg AGE·g^-1^ dry weight for the untreated samples. The results obtained in this study were similar to those reported by Milledge et al. [7] and Davis et al. [34] for *Sargassum* spp. After pretreatment, TPC showed significant differences (*p* <0.001). The S+W and CS pretreatments achieved reductions of 19 and 64%, respectively. The results obtained using CS can be attributed to the use of formaldehyde, which binds phenolic compounds [35]. The difference in UT biomass was not significant only in the case of WW pretreatment (*p* = 0.2794076).

### 3.2 Effect of pretreatments on mineral content

Table 2 shows the mineral content, mainly light metal ions of Na, K, Ca, and Mg, present in untreated and pretreated *Sargassum* biomass. The UT samples presented a high content of Ca, followed by Na and K, and to a lesser extent, Mg. This result is similar to those reported by Davis et al. [34] and Rodrígez-Martínez et al. [1], who also found a high content of Ca, followed by K, Na, and Mg. The total mineral content in this study was 125.4 mg·g^-1^, with Ca contributing 60% of the total mineral content. With the application of pretreatments, a significant reduction in the total content of these minerals was achieved (*p* <0.001).

**Table 2:** Influence of pretreatments on the content of mineral salts and total removal in the biomass of *Sargassum* spp. UT = Untreated, WW = Water washing, S+W = Soaking + Warm water washing, CS = Chemical soaking.

	Na	K	Ca	Mg	Total*	Removal
	mg·g^-1^ dry weight					%
**UT**	32 ±0.2	10.9 ±1.2	74.8 ±2.5	7.7 ±0.9	125.4	
**WW**	23.4 ±0.9	7.9 ±0.1	48.1 ±1.9	8.9 ±0.2	88.3	30
**S+W**	6.6 ±0.5	2.0 ±0.09	70.8 ±1.3	10.8 ±0.3	90.2	28
**CS**	4.1 ±0.06	0.4 ±0.1	46.9 ±1	2.8 ±0.13	54.2	57

Data represent mean ± SD, n = 2.

*The data sets were analyzed with a one-way ANOVA showing a *p* <0.001.

The CS pretreatment achieved the most significant reduction in minerals (Na, K, Ca, and Mg) compared to UT biomass (*p* <0.001), which represented a removal of 57%. Ross et al. [18] also reported removals of Mg, K, Na, and Ca in brown algae of up to 90% with the use of an acid pretreatment, while water pretreatment achieved reductions of 30–40%. The pretreatment S+W results are better than the pretreatment WW for reducing Na and K content, which is advantageous because they have been reported to govern the toxicity of methanogens [12]. However, WW and S+W did not present significant differences, as both achieved a similar reduction in minerals.

### 3.3 Effect of pretreatments on the theoretical energy content of *Sargassum* spp.

The TBMP of the untreated samples was 335.8 mL CH_4_·g^-1^ VS, and after pretreatment, the TBMP improved by up to 50%, as shown in Table 1. The increase in TBMP appears to be associated with the reduction of ash and minerals in algal biomass. Table 1 shows an increase in the percentages of organic elements, mainly C and H, after pretreatments. This change in the elemental composition modifies the stoichiometric formula and, thus, the amount of methane that can be predicted. The CS pretreatment reached the highest value, possibly because of the removal of inorganic matter with the use of chemical solutions. Hessami et al. [36] and Mhatre et al. [26] also found an increase in the TBMP after the removal of some bioproducts using different acidic and alkaline solutions. On the other hand, the HHV of biomass doubled after pretreatments, with the CS pretreatment achieving a value 2.4 times higher than that of the untreated *Sargassum* biomass. These results are attributed to the removal of inorganic materials, such as ash [37].

### 3.4 Effect of pretreatments on the biomethane potential of *Sargassum* spp.

The average values obtained for the accumulated biogas, methane, and biodegradability indices for each pretreatment are listed in Table 3. After 53 days, the BMP of the cellulose (CL) used as a positive control produced 374.4 ±7.5 mL CH_4_·g^-1^ VS, which validates the results because it fits the criteria established by Holliger et al. [23].

**Table 3:** A summary of the results of the biomethane potential test of untreated and pretreated *Sargassum* spp. after 70 days of incubation and 53 days for cellulose.

Substrate	Biogas Yield (N mL·g^-1^ VS)	Methane content (%)	Methane yield* (N mL·g^-1^ VS)	BI (%)	Pretreatment efficiency (%)
**CL**	578.6 ±18.9	55.7	374.52 ±7.5	90.2**	-
**UT**	131.53 ±3.7	61.7	77.64 ±3.9	23.09	-
**WW**	193.19 ±15.6	56.0	107.42 ±6.7	32.11	+38
**S+W**	176.27 ±7.5	54.9	97.75 ±5.4	29.08	+26
**CS**	150.4 ±5.4	49.1	74.77 ±5.4	22.23	-4

CL = Cellulose, UT = Untreated, WW = Water washing, S+W = Soaking + Warm water washing, CS = Chemical soaking.

* *p-*value = 0.000121.

**BI according to the TBMP of cellulose Filer et al. [38].

**Fig 2:**
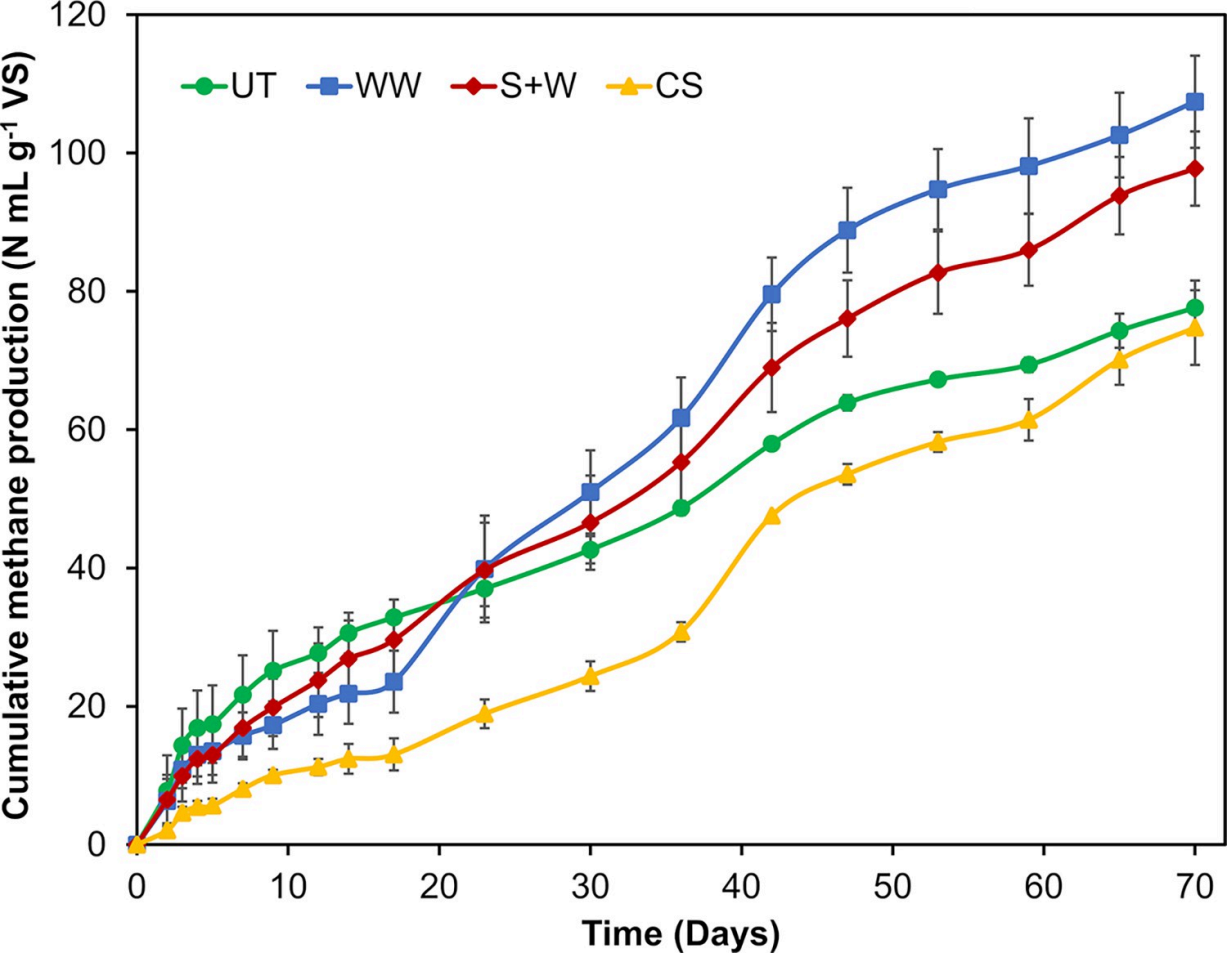
Cumulative methane production expressed in N mL·g^-1^ VS of pretreated and untreated *Sargassum* spp. biomass after 70 days of incubation. UT = Untreated, WW = Water washing, S+W = Soaking + Warm water washing, CS = Chemical soaking. Error bars represent the standard deviation of the mean (n = 3).

In contrast to the control, all experiments stabilized and reached the final methane production after 70 days of incubation. The net cumulative methane production is shown in Fig 2.

The methane production of the UT biomass was 77.64 ±3.9 mL CH_4_·g^-1^ VS. Pretreatments had a significant effect on the BMP (*p* <0.001). The best result was obtained with WW pretreatment, which generated a BMP of 107.42 ±6.7 mL CH_4_·g^-1^ VS, which represented an increase of 38% compared to the UT biomass. The S+W pretreatment resulted in a result close to that of WW, which represented an increase of 26%. However, the BMP between the WW and S+W pretreatment groups was statistically similar (*p* = 0.1706440). Yields similar to those achieved in this study have been reported by Tapia-Tussell et al. [9] and Thompson et al. [10]. In contrast, CS pretreatment, despite having the best increase in TBMP and presenting the best reduction in inorganic matter and total phenol content in the biomass, negatively affected BMP. The CS generated the lowest BMP, representing a decrease of 4%. CS did not show significant differences compared to UT (*p* = 0.8891660).

WW and S+W pretreatments succeed only in boosting methane yield; however, they did not improve the *Sargassum* degradation rate, as the duration of the BMP assay was longer than that observed in previous studies [7, 9, 10]. Furthermore, the methane production curves (Fig 2), present a staggered shape over time, indicating that the hydrolysis of the material is the limiting step [38]. This was reflected in the biodegradability index, because the values achieved were less than 50% (Table 3). Therefore, studies focusing on improving the hydrolysis of *Sargassum* spp. biomass are required.

**Fig 3:**
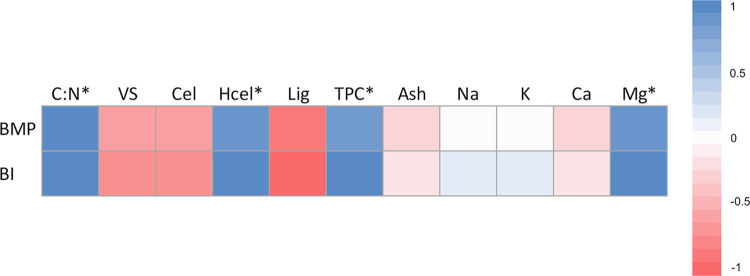
Heat map of Spearman’s correlation analysis between the compositional characteristics of *Sargassum* spp. and BMP/BI. Cel = Cellulose; Hcel = Hemicellulose; Lig = Lignin; TPC = Total phenolic content. The mark * indicates the significance test (*p* <0.05).

As shown in Fig 3, a strong positive correlation was observed between the C: N ratio and the BMP (r = 0.79, *p* <0.01) as well as with the BI (r = 0.80, *p* <0.01). This result indicates that a higher C: N ratio in the substrate promotes increased methane generation and biodegradability and supports the notion that increased carbon content compared to nitrogen is favorable for methanogenic microorganisms [7]. Similarly, hemicellulose, total phenols, and magnesium had strong positive correlations with BMP and BI (*p* <0.01), indicating that these specific components play crucial roles in enhancing biomethane production and biodegradability. Hemicellulose, being a complex carbohydrate in plant cell walls, is known to be readily fermentable and could contribute to increased methane production. Phenolic compounds are described as inhibitors of anaerobic digestion, however in this study, it appears that they had no effect on methane yield possibly because the phenol content was lower than that reported by other authors [7, 9]. Magnesium, an essential element involved in various biological processes. Mg is an important component in the synthesis of biological macromolecules and acts as an activator of biological enzymes [13], suggesting its potential role in promoting methanogenesis.

Other variables, such as mineral salts, VS, ash, cellulose, and lignin, showed no significant correlations with BMP and BI. However, it is crucial to emphasize that the absence of strong correlations does not imply that these variables do not influence the process. Other elements or interactions not explored in this study may have an impact on methane generation and biodegradability.

The methane yields achieved in this study were similar to those reported by other authors [9, 10, 39]; however, our results were obtained using more economical and less energy-consuming methods. This technology requires lower energy input than other physical pretreatment methods and eliminates the need for expensive chemicals and enzymes. The chemical treatment tested in this study (CS) could be considered a more sustainable and cost-effective method because of its milder reaction conditions. However, the use of HCl and formaldehyde is costly to acquire and manage, and their use raises safety and environmental concerns. Overall, water washing pretreatment is a straightforward and environmentally beneficial approach for removing pollutants from algal biomass, making it suitable for downstream processing. S+W also generated positive results and can be considered an economical method, similar to WW, because it uses water as the main washing medium. The disadvantage is that it requires a residence time of 24 h and energy to heat water from room temperature to 40°C. One potential disadvantage of WW pretreatment is the use of freshwater, because it is a natural resource and its responsible use should be considered.

### 3.5 Microbial community analysis

Analysis of the microbial community structure was conducted on the WW and CS reactors and the inoculum. Alpha-diversity indices that reflect diversity, richness, and evenness were measured using the Shannon and Simpson indices and a richness estimator, Chao 1 (Table 4). The high numerical values of bacterial sequences and ecological indices indicated that bacterial diversity and richness greatly exceeded those of archaea; similar results have been previously reported [40].

**Table 4:** Alpha-diversity indices of the different microbial communities. WW = Water washing, CS = Chemical soaking.

		Sequence number	Shannon	Simpson	Chao 1
**Bacteria**	Inoculum	145,368	4.46	0.84	390
	WW	163,129	4.50	0.85	356
	CS	161,928	4.19	0.82	339
**Archaea**	Inoculum	2,035	2.12	0.65	10
	WW	1,554	2.64	0.78	12
	CS	2,983	2.77	0.79	15

The Shannon index provides a quantitative measure of the species richness and evenness [41]. In the bacterial community, this value decreased in the CS system compared to that in the inoculum and WW systems, indicating that diversity and richness decreased. In contrast, the archaeal communities in the WW and CS systems showed an increase in the Shannon index with respect to the inoculum, indicating an increase in the diversity and richness of both communities. These results for the bacterial and archaeal communities were also reflected in the Chao1 estimator, which is a nonparametric method for estimating the number of species in a community [42].

Simpson’s indices were similar among the bacterial communities ranging from 0.82 to 0.85. A lower value suggests that the microbial community is more diverse and evenly distributed, whereas a higher value suggests that the microbial community is dominated by a few abundant taxa [42]. Therefore, these values indicate that the bacterial communities had little evenness in the population size of each of the taxa present, that is, there were taxa with higher relative abundances. The archaeal community present in the WW and CS systems showed an increase in the Simpson index with respect to the inoculum, indicating a decrease in the evenness of the populations. However, the dominant taxa assigned to Bacteria and Archaea in the inoculum source remained the same during AD of *Sargassum* spp.

**Fig 4:**
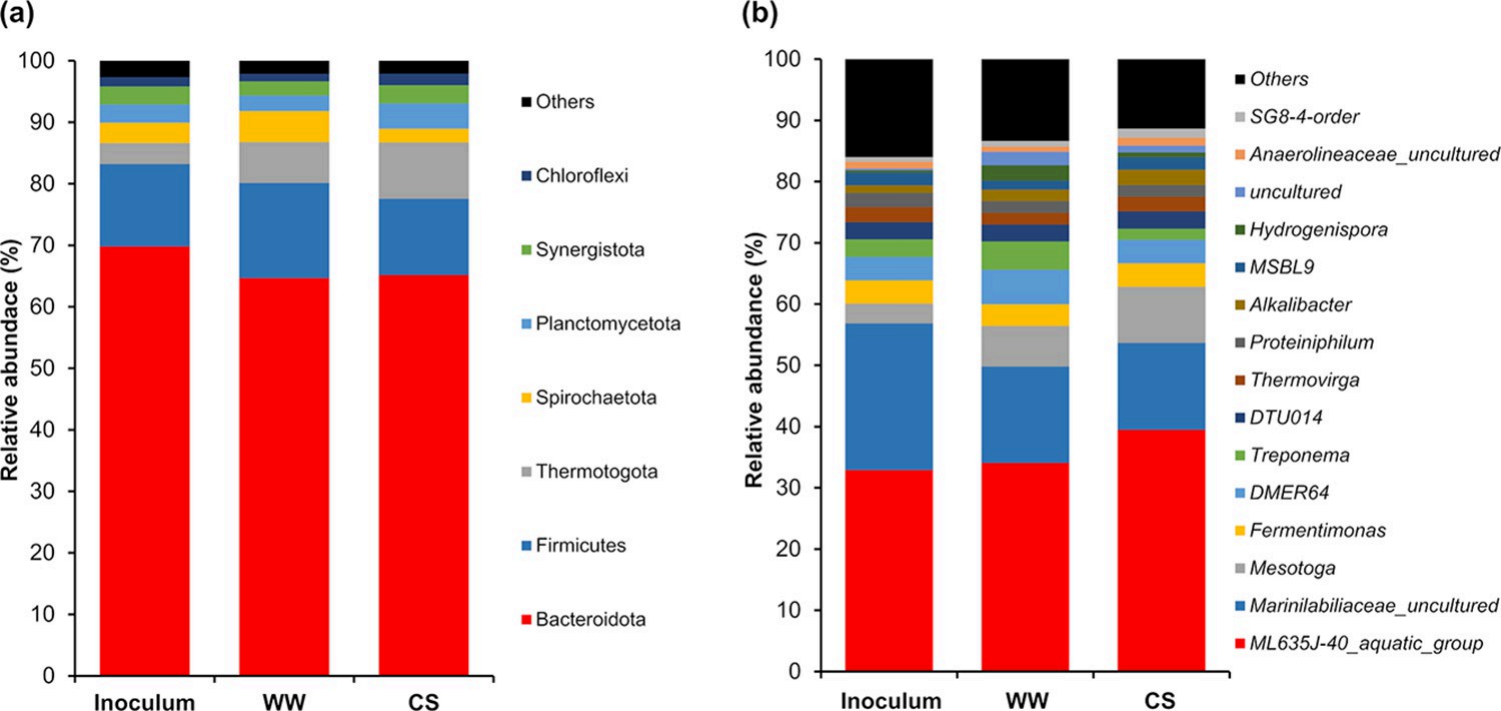
Relative abundances of bacterial communities at the phylum (a) and genus (b) level of the inoculum, WW (water washing), and CS (chemical soaking). Taxa with abundances <1% of the total sequences in the samples were grouped as other.

The relative abundance of the bacterial communities at the phylum and genus levels is shown in Fig 4. In both the inoculum and the WW and CS reactors, no differences in dominant taxa were observed at the phylum levels (Fig 3A). All three systems were dominated by Bacteroidota (64–69%), Firmicutes (12–15%), Thermotogota (3–9%), Planctomycetota (2–4%), Synergistota (2–3%), Spirochaetota (2–5%), and Chloroflexi (1–2%).The microorganisms assigned to these taxa convert complex macromolecular organic compounds into biodegradable molecules [43]. Therefore, these taxa are usually present during hydrolysis and acidogenesis. The phylum Firmicutes also represents syntrophic bacteria that are involved in the acetogenic phase [43]. Studies have shown that members of the phylum Bacteroidota are dominant in systems with readily biodegradable feedstocks [21]. However, the biomass of *Sargassum* spp. is characterized by a low availability of biodegradable compounds. Therefore, the dominance of the phylum Bacteroidota in the WW and CS systems was attributed to the inoculum source, which was fed with the liquid fraction of RSOM, which is characterized by high biodegradability.

As shown in Fig 4B, at the genus level, *ML635J-40_aquatic_group* dominated in both the inoculum (32%) and WW (34%) and CS (39%) systems. This group of microorganisms was assigned to the ML635J-40_aquatic_group family, which is characterized by the fermentation of organic materials, such as sugars, starches, or more complex polysaccharides [44]. Another group of bacteria, classified as *Marinilabiliaceae_uncultured*, belonging to the Marinilabiliaceae family, was abundant in the inoculum (24%). However, a decrease was observed in the WW (16%) and CS (14%) systems. Members of this family are characterized by being saccharolytic, that is, they ferment sugars [45]. Therefore, we can attribute the decrease in Marinilabiliaceae to the scarcity of fermentable sugars in CS and WW media. In contrast, an increase in the abundance of the genus *Mesotoga* was observed in WW (7%) and CS (9%) compared to the inoculum (3%). This genus is abundant in mesothermal anaerobic environments rich in aromatic compounds. It can perform H_2_ oxidation and thiosulfate reduction using elemental sulfur as the final electron acceptor [46]. Therefore, the slight increase in *Mesotoga* can be attributed to the presence of aromatic compounds derived from the decomposition of lignocellulosic material in the WW and CS. In addition, an increase in the *DMER64* genus was observed in WW (6%) compared to CS (4%) and inoculum (4%). Bacteria of this genus are hydrogen (H_2_) producers. The genus *DMER64* can establish direct electron transfer (DIET) with methanogens during VFA degradation [47]. The genera Treponema and Hydrogenispora were dominant in the WW system. Treponema is probably a homoacetogenic, saccharolytic spirochete, that interacts with cellulolytic bacteria and consumes H_2_ and CO_2_ to produce acetate [48]. The genus *Hydrogenispora* comprises H_2_-producing and spore-forming bacteria. It belongs to the bacterial phylum Firmicutes, which plays an essential role in the biodegradation of substrates, with a rapid hydrolysis rate and high concentrations of ammonia [47]. According to the results, the genera *Marinilabiliaceae_uncultured*, *DMER64*, *Treponema*, and *Hydrogenispora* can be related to high methane production due to their dominance in the WW system.

**Fig 5:**
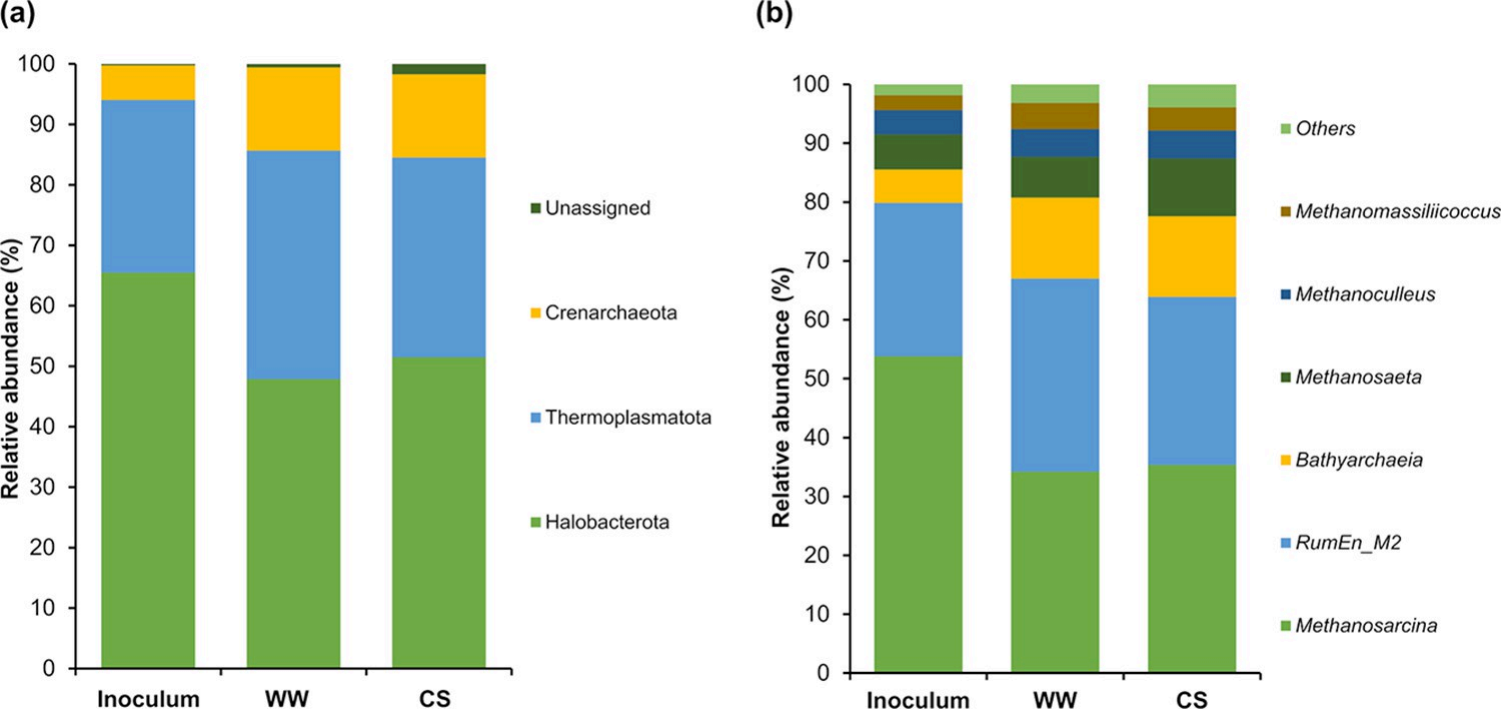
Relative abundances of archaeal communities at the phylum (a) and genus (b) level of the inoculum, WW (water washing), and CS (chemical soaking). Taxa with abundances <2% of the total sequences in the samples were grouped as other.

The results of the analysis of archaeal community structure at the phylum and genus levels are shown in Fig 5. Most of the 16S rRNA gene reads from both the inoculum and WW and CS were assigned to the phyla Holobacterota (47–65%), Thermoplasmatota (28–37%), and Crenarchaeota (6–13%). Currently, Halobacterota and Thermoplasmatota contain most known methanogens.

At the genus level, *Methanosarcina*, *RumEn_M2*, *Bathyarchaeia*, *Methanosaeta*, *Methanoculleus*, and *Methanomassiliicoccus* were detected (Fig 5B). These genera are known to play important roles in methanogenesis, except for *RumEn_M2* and *Bathyarchaeia*. Until recently, there was little information on their involvement in methanogenesis. The genus *RumEn_M2* (family Methanomethylophilaceae) is now known to be a new methanogenic genus, which is restricted to growth on methanol and methylamines and is strictly H_2_-dependent [49]. In addition, the presence of putative methane-metabolizing genes in members of Bathyarchaeia suggests that methanogenesis may be more widespread phylogenetically [50].

*Methanosarcina*, which are mixotrophic methanogens and can metabolize acetate, hydrogen, and C_1_ compounds, were dominant in the inoculum (54%). However, the relative abundance of *Methanosarcina* decreased in the WW (34%) and CS (35%) systems, despite being characterized by high tolerance to stress conditions and environmental changes. This decrease can be attributed to the low acetogenic activity in the WW and CS media. High concentrations of acetate favor the growth of *Methanosarcina* [51]. Therefore, there was a slight increase in the genus *Methanosaeta* in WW (7%) and CS (10%) compared with the inoculum (6%), which is usually more abundant at low acetate concentrations because of its high affinity for the substrate. However, an increase in the relative abundance of *Bathyarchaeia* from 6% (inoculum) to 14% was observed in the WW and CS. Li et al. [52] reported that *Bathyarchaeia* can perform the same functions as some hydrogenotrophic methanogens and therefore displace them due to their high affinity for H_2_ and CO_2_. The ability of *Bathyarchaeia* to reduce the abundance of *Methanosarcina*, which was dominant in the inoculum but decreased in abundance in the WW and CS after *Bathyarchaeia* increased. The genus *Methanomassiliicococcus*, a methylotrophic methanogen, also increased in abundance during *Sargasum* AD and was dominant in the WW system. These results showed that *Sargassum* spp. decreased the activity of the acetoclastic pathway and increased that of the hydrogenotrophic pathway. This may be due to the low number of fermentable compounds by the hydrolytic bacteria that reduced the activity of the acidogenic and acetogenic bacteria, due to the low biodegradability of *Sargassum* spp. According to these results, the high methane production can be attributed to the genera *Rumen-M2*, *Bathyarchaeia*, and *Methanomassiliicoccus*, which were dominant in the WW system.

As described above, CS and WW had contrasting compositions in terms of their C: N ratios, mineral content, and lignin content. Differences in their compositions may have influenced the microbial communities and their metabolic activities. CS, with its low C: N ratio, may have restricted the availability of organic carbon and the essential nutrients required for methane production. It also had a higher lignin content and a more recalcitrant and difficult substrate for microbial degradation. This could also lead to a decrease in the bacterial diversity and richness in the CS system, as indicated by the Shannon index results. In contrast, WW had a higher C: N ratio and lower lignin content, which may have provided a more favorable environment for microbial activity and methane production. The increased diversity and richness of bacteria and archaea in the WW system, as indicated by the Shannon index results, suggest a more conducive environment for microbial growth.

## 4. Conclusions

Pretreatments successfully modified the composition of Sargassum spp. WW had a positive effect on the C: N ratio, which was strongly correlated with the methane yield. CS pretreatment was the best for the removal of mineral salts and for the improvement of the theoretical energy content; however, it did not improve the experimental methane yield. While it is important to increase the C: N ratio and reduce the inorganic compounds in *Sargassum* spp. biomass, the presence of insoluble fibers continues to be a barrier to biodegradability and methane production. The cellulose, hemicellulose, and lignin contents did not decrease after pretreatments, highlighting the need for pretreatments that focus on enhancing the hydrolysis of lignocellulosic biomass.

The composition of *Sargassum* spp. influenced the microbial community composition and methane production. The presence of genera *Marinilabiliaceae_uncultured*, *DMER64*, *Treponema*, *Hydrogenispora Rumen-M2*, *Bathyarchaeia*, and *Methanomassiliicoccus* could be associated with the high methane yield of *Sargassum* spp. since they presented the highest relative abundance in the WW system. However, the microbial community composition involved in AD of *Sargassum* spp. seems to depend on the inoculum source. Overall, this study suggests that water washing is essential for pretreatment of algal biomass. However, freshwater is a valuable and limited resource, so it is necessary to explore alternative water sources for algal pretreatment, such as non-potable water, brackish water, or recycled water from other processes.

## Supporting information

S1 Data (XLSX)

S2 Data (XLSX)
